# Anti-Inflammatory and Antioxidant Mechanisms of *Dendrobium moschatum* Polysaccharide in Intestinal Epithelial Cells via TLR4-NF-κB and Nrf2 Signaling Pathways

**DOI:** 10.3390/antiox14111384

**Published:** 2025-11-20

**Authors:** Ji Chen, Chunyan Ma, Xu Mo, Linhong Li, Lijuan Wu, Chaowen Zhang, Rui Li, Yuanfeng Zou, Fan Liu, Mengliang Tian

**Affiliations:** 1 College of Agronomy, Sichuan Agricultural University, Wenjiang 611130, China; cma731465@gmail.com (C.M.); 2020301094@stu.sicau.edu (X.M.); wulijuan0921@126.com (L.W.); 71352@sicau.edu.cn (R.L.); 10060@sicau.edu.cn (F.L.); 2 Leshan Academy of Agricultural Sciences, Leshan 614000, China; llhworks@163.com; 3 Natural Medicine Research Center, College of Veterinary Medicine, Sichuan Agricultural University, Wenjiang 611130, China; zhangchaowen1@stu.scu.edu.cn (C.Z.); yuanfengzou@sicau.edu.cn (Y.Z.)

**Keywords:** *Dendrobium moschatum* polysaccharides, intestinal epithelial cells, structural characterization, antioxidant activity, anti-inflammatory effect, LPS-induced injury

## Abstract

*Dendrobium moschatum* neutral polysaccharide (DMP-NP) was isolated using a water extraction–ethanol precipitation method, followed by purification with DEAE-cellulose anion-exchange resin and a dextran gel column. The resulting DMP-NP1 exhibited a weight-average molecular weight of 16.23 kDa. The molar ratio of monosaccharides was as follows: glucose–mannose–galactose–fucose–rhamnose = 78.54:19.11:1.59:0.53:0.23, with a glucose-to-mannose ratio of 4.1:1. Infrared spectroscopic analysis revealed characteristic carbohydrate absorption peaks and confirmed the presence of pyranosidic linkages. NMR analysis revealed that DMP-NP1 possesses a backbone mainly formed by 1→4 glycosidic linkages, a small number of 1→6 branches, and O-acetyl substitutions at the C2 and C3 positions of mannose residues. In vitro experiments demonstrated that treatment with 0–20 μg/mL (0–1.23 μM) DMP-NP significantly enhanced the activities of catalase (CAT) and superoxide dismutase (SOD) in IPEC-J2 cells, along with upregulation of the corresponding antioxidant genes. Concurrently, DMP-NP reduced the secretion of key pro-inflammatory cytokines, including TNF-α, IL-1β, and IL-6, and downregulated the expression of genes associated with both antioxidant and inflammatory signaling pathways. Collectively, these findings indicate that DMP-NP not only prevents but also ameliorates LPS-induced inflammatory injury in intestinal epithelial cells, thereby providing a basis for the application of DMP-NP in intestinal inflammation mitigation.

## 1. Introduction

Inflammatory bowel disease (IBD), a chronic gastrointestinal disorder characterized by abdominal pain, diarrhea, and progressive intestinal damage, has emerged as a global health challenge with rising incidence and limited therapeutic options [1]. While current clinical treatments, including corticosteroids, immunosuppressants, and biologics, can alleviate symptoms, prolonged use frequently results in severe complications such as osteoporosis, metabolic disorders, and gastrointestinal dysfunction [2]. Consequently, the development of natural plant-based drugs with high efficacy and low toxicity is an urgent area of research. Among these, polysaccharides from *Dendrobium* species, a genus of medicinal orchids, have shown remarkable potential in mitigating inflammation-related pathologies.

*Dendrobium*, a highly valued medicinal plant from the Orchidaceae family, is celebrated for its bioactive compounds, with polysaccharides serving as a key active ingredient. These polysaccharides, large biomolecules composed of over 10 monosaccharides linked by glycosidic bonds, exhibit a wide range of pharmacological activities, including immune modulation, anti-tumor effects, antioxidant properties, and anti-hyperglycemic effects [3]. In addition, dendrobine—another major bioactive constituent of Dendrobium—has been reported to exert notable anticancer effects, particularly in renal cell carcinoma [4]. For example, polysaccharides from *Dendrobium huoshanense* have demonstrated significant anticancer potential [3], while those from *Dendrobium officinale* alleviate secondary pulmonary injury induced by colitis through the inhibition of TLR4 signaling and activation of the Nrf2 pathway [5]. Moreover, water-soluble polysaccharides from *Dendrobium hancockii* have shown antioxidant properties [6]. Despite the therapeutic potential of *Dendrobium* polysaccharides, current research predominantly focuses on a few cultivated species (e.g., *D. officinale*), leaving the majority of *Dendrobium* resources, particularly rare species like *D. moschatum*, underexplored [7]. Moreover, the absence of a systematic structure–activity relationship (SAR) analysis limits the development of polysaccharide-based therapies for inflammatory bowel disease (IBD). Advancing research on rare species and establishing a structure–activity correlation model for these polysaccharides is essential not only for furthering the theoretical understanding of medicinal plants but also for broadening the application of *Dendrobium* resources.

Notably, *D. moschatum*, a species traditionally revered for treating gastrointestinal ailments in Southeast Asia, has been extensively studied for its lignan components (e.g., moschatum), but its polysaccharide fraction (DMP-NP) remains underexplored. Our study suggests that *D. moschatum* polysaccharides may possess a unique monosaccharide profile dominated by glucose and mannose (4.1:1 ratio), which could contribute to enhanced solubility and receptor binding compared to other *Dendrobium* species. However, critically, the precise chemical structure of DMP-NP and its mechanism of action in intestinal inflammation remain entirely elusive.

This study aims to characterize the chemical structure of DMP-NP1, elucidate its anti-inflammatory and antioxidant mechanisms, with a focus on the TLR4-NF-κB and Nrf2 signaling pathways, in intestinal epithelial cells, and establish a structure–activity relationship model to guide the development of polysaccharide-based IBD therapeutics. This work represents the first comprehensive analysis of the structure and mechanisms of DMP-NP1, bridging the gap between traditional medicinal uses and modern pharmacological validation. Our findings not only advance the clinical translation of *Dendrobium* polysaccharides for IBD treatment but also offer a valuable framework for accelerating the development of underutilized medicinal plant resources.

## 2. Materials and Methods

### 2.1. Materials

Fresh *D. moschatum* stems were cleaned, dried, and stored in a dry environment for later use. *D. moschatum* was collected from Mangshi, Dehong Prefecture, Yunnan Province, and identified as *D. moschatum*. Mature stems from three-year-old plants were harvested during the harvest season in November for experimental use. The samples underwent washing, and thermal treatment at 105 °C for 30 min, drying at 50 °C until a constant weight was achieved, crushing, sieving through a 60-mesh sieve, and were then stored in a dry environment for further use.

DEAE-cellulose and dextran gel columns, as well as ELISA kits, were purchased from Ruidahenghui Technology Development Co., Ltd., Beijing, China. The CCK-8 kit and dialysis bags with a molecular weight cut-off of 3500 Da were obtained from Solarbio, Beijing, China. All other analytical-grade reagents were purchased from Kelong Chemical Co., Ltd., Chengdu, China.

### 2.2. Isolation and Purification of D. Moschatum Polysaccharides

A total of 60 g of *D. moschatum* powder was placed in a 2000 mL round-bottom flask, and 1200 mL of 95% ethanol solution was added for reflux extraction. This extraction was performed four times for 2 h each. After each extraction, the solution was filtered through a 300-mesh filter to remove the liquid and obtain the *Dendrobium* powder. The resulting defatted powder was thoroughly dried at 50 °C and then subjected to hot water extraction (80 °C, 2 h) twice, using 40 volumes of distilled water each time, to solubilize the polysaccharides. The filtrates were combined, concentrated to approximately 100 mL using a rotary evaporator, and then 4 times the volume of anhydrous ethanol was added. The mixture was kept at 4 °C overnight. The precipitate was collected by centrifugation or filtration, followed by freeze-drying to obtain crude polysaccharides, which were stored at −20 °C.

The crude polysaccharides were dissolved in distilled water and dialyzed using a dialysis bag with a molecular weight cut-off of 3500 Da to remove small molecules and salts. After dialysis, the solution was freeze-dried to obtain dried polysaccharides. A 200 mg sample of the dried polysaccharide was dissolved in 20 mL of distilled water and filtered using a 0.45 μm water-based mixed cellulose ester (MCE) membrane filter. The clarified solution was loaded onto a DEAE-cellulose column and eluted with distilled water at a flow rate of 2 mL/min. Fractions were collected automatically at 5 min intervals. The polysaccharide content in each fraction was determined using the phenol–sulfuric acid method, and the elution curve was plotted to analyze the polysaccharide content in each fraction. Fractions corresponding to the major peak in the elution profile were pooled, lyophilized, and designated as the purified neutral polysaccharide DMP-NP1.

### 2.3. Molecular Weight Determination of DMP-NP

The weight-average molecular weight of DMP-NP1 was determined by gel permeation chromatography coupled with multi-angle light scattering (GPC-MALS). The system consisted of a Thermo U3000 HPLC system (Thermo Fisher Scientific, Walthan, MA, USA) and a Wyatt DAWN HELEOS II MALS detector (Wyatt Technology, Santa Barbara, CA, USA). Purified DMP-NP1 (5 mg) was dissolved in 1 mL of 0.1 M sodium nitrate to yield a 5 mg/mL stock solution. Polyethylene oxide (PEO) solution with a molecular weight of 18.67 kDa was used as the standard. Chromatographic separation was performed under the following conditions: 100 μL injection volume, 0.9% (*w*/*v*) NaCl mobile phase, 0.5 mL/min flow rate, and a column temperature maintained at 30 °C. The molecular weight of the sample was analyzed and determined using the GPC-MALS system by comparison with the standard.

### 2.4. Chemical Composition and Monosaccharide Composition Analysis

A 1 mg/mL aqueous solution of DMP-NP1 was prepared and subjected to compositional analysis: total carbohydrate content by the phenol–sulfuric acid method [8], total phenolic content by the Folin–Ciocalteu assay [9], and total protein content by the Coomassie Brilliant Blue G-250 method [10].

Monosaccharide composition was analyzed by PMP derivatization high-performance liquid chromatography (HPLC) [11]. A 20 mg sample of DMP-NP1 was dissolved in 1 mL of distilled water, followed by the addition of 200 μL of 0.6 mol/L NaOH and 200 μL of 0.5 mol/L PMP. The derivatization was carried out at 70 °C for 1 h and then quenched with 400 μL of 0.3 M HCl. The resulting solution was subjected to repeated chloroform extractions, followed by centrifugation (3000 rpm, 5 min each), until the organic phase became colorless. The aqueous phase was retained as the sample. The solution was filtered through a 0.22 μm micropore filter membrane and analyzed using an Agilent 1260 series HPLC system (Agilent Technologies, Santa Clara, CA, USA) with a ZORBAX SB-C18 column (250 mm × 4.6 mm, 5 μm, Agilent Technologies, Santa Clara, CA, USA). Gradient elutionwas performed using acetonitrile (A) and 0.05 mol/L phosphate buffer (pH 6.7) (B).

### 2.5. FT-IR and NMR Analyses

For FT-IR analysis, 1 mg of dried DMP-NP1 was thoroughly blended with potassium bromide, finely ground using an agate mortar, and compressed into transparent pellets. Spectra were acquired in the range of 4000–400 cm^−1^ on a Fourier transform infrared (FT-IR) spectrometer (Tensor 27, Bruker, Karlsruhe, Germany).

For NMR analysis, DMP-NP1 was repeatedly exchanged with D_2_O (Sigma-Aldrich, St. Louis, MO, USA) by dissolving and freeze-drying (three cycles). NMR spectra, including ^1^H, ^13^C, HSQC, and HMBC, were recorded on a Bruker Avance III HD 800 MHz spectrometer (Bruker, Karlsruhe, Germany) operating at 60 °C, using sodium 2,2-dimethyl-2-silapentane-5-sulfonate (DSS) as an internal reference. All spectra were processed and analyzed using MestReNova software 15.0.0-34764 (Mestrelab Research, Santiago, Spain).

### 2.6. Anti-Inflammatory and Antioxidant Activity of D. Moschatum Polysaccharides on Intestinal Epithelial Cells (IPEC-J2)

#### 2.6.1. Cell Culture

The cells used in our study were obtained from the Natural Medicine Research Center, College of Veterinary Medicine, Sichuan Agricultural University. Cryopreserved cells were rapidly thawed in a 37 °C water bath and centrifuged at 1000 rpm for 5 min. The pellet was resuspended in 1 mL of complete medium, transferred to a culture dish containing 9 mL of fresh medium. The supernatant was discarded, and the cells were resuspended in 1 mL of complete culture medium and transferred to a culture dish containing 9 mL of complete culture medium. The cells were dispersed by shaking in a crosswise motion and incubated in a 37 °C, 5% CO_2_, and 90% humidity incubator. When the cells reached confluence, the culture medium was discarded, and the cells were washed twice with 4 mL of PBS buffer. Then, 2 mL of PBS buffer and 1 mL of trypsin-EDTA solution were added to digest the cells for 3 min. Following the addition of 4 mL of DMEM to quench the trypsinization, the cells were mechanically dislodged by gentle pipetting. The resulting cell suspension was then centrifuged at 1000 rpm for 5 min, and the pellet was resuspended in 2 mL of complete culture medium.

Cell toxicity of DMP-NP1 was assessed using the CCK-8 assay. Cells were seeded into 96-well plates at 100 μL per well and incubated for 12 h. After aspirating the medium, the cells were treated with a range of DMP-NP1 concentrations (5–100 μg/mL; corresponding to 0.31–6.16 μM) for 24 h. After adding 10 μL of CCK-8 solution to each well, the cells were incubated for 1 h, and absorbance at 450 nm was measured.

#### 2.6.2. Prevention and Treatment of LPS-Induced Inflammation in IPEC-J2 Cells

IPEC-J2 cells were seeded into 6-well plates and cultured for 24 h. Subsequently, the culture medium was aspirated, and the cells were washed twice with PBS. Then, the cells were treated with DMP-NP1 for 24 h, followed by 1 mL of PBS washing twice. LPS (20 μg/mL) was then added for 24 h. The experimental design comprised the following groups: a blank control (medium only), an LPS-induced model group (20 μg/mL), and LPS + DMP-NP1 co-treatment groups (0.31, 0.62, and 1.23 μM). After two PBS washes, cells were lysed mechanically, and the lysates were centrifuged at 1000 rpm for 10 min at 4 °C. The resulting supernatants were collected, and the concentrations of TNF-α, IL-1β, IL-6, SOD, and CAT were quantified using commercial ELISA kits according to the manufacturers’ protocols. For gene expression analysis, total RNA was extracted from the cells using the TRIzol method. The extracted RNA was reverse-transcribed into cDNA, and the expression levels of inflammation-related and antioxidant pathway genes were measured by RT-qPCR. To evaluate protective efficacy, cells were pretreated with DMP-NP1 prior to LPS challenge. To assess therapeutic potential, LPS was administered first, followed by DMP-NP1 treatment.

### 2.7. Quantitative Real-Time PCR Analysis

Total RNA was extracted using TRIzol reagent and reverse-transcribed into complementary DNA (cDNA). Quantitative real-time PCR (qRT-PCR) was performed using a fluorescence quantitative PCR master mix from Yeasen Biotechnology to configure a 10 μL reaction mixture. The qRT-PCR protocol was as follows: initial denaturation at 95 °C for 30 s; followed by 39 cycles of denaturation at 95 °C for 5 s and annealing/extension at 59.6 °C for 30 s. A melt curve analysis was subsequently conducted: 95 °C for 10 s, 65 °C for 5 s, and then a gradual increase to 95 °C at a ramp rate of 0.5 °C per minute. All primers were synthesized by Tsingke Biotechnology Co., Ltd. (Beijing, China). The primer sequences were as follows: β-Actin: Forward 5′-GATGAGATTGGCATGGCTTT-3′, Reverse 5′-CACCTTCACCGTTCCAGTTT-3′; IL-6: Forward 5′-TAACCCCACCACAAATGCC-3′, Reverse 5′-CCCCAGTACATTCTCCGAA-3′; IL-1β: Forward 5′-ATGGCTAACTACGGTGACAAC-3′, Revers-e 5′-CCATCAGCCTCAAATAACAGGT-3′; TNF-α: Forward 5′-TTGAGCATCCAACCCTCTGGC-3′, Reverse 5′-CCATCAGCCTCAAATAACAGGT-3′; SOD: Forward 5′-CACCATCTACTTCGAGCTG-3′, Reverse 5′-AGCCTTGTGTATTATCTCC-3′; CAT: Forward 5′-TGTGAACTGTCCCTTCCGTG-3′, Reverse 5′-CGTCTGTTCGGGAGCACTAA-3′; GPX1: Forward 5′-CGGACCACCTGTTGAAAGCTC-3′, Reverse 5′-TCCGCCAGTTCTTGTTGTCCA-3′; Nrf2: Forward 5′-CACCACCTCAGGGTAATA-3′, Reverse 5′-GCGGCTTGAATGTTTGTC-3′; KEAP1: Forward 5′-TACACGCCATGTGTTTGAACT-3′, Re-verse 5′-CCTTCTTCGCCATTCTCAGC-3′; HO-1: Forward 5′-GGCCTTGTATAATCCCTGAT-3′, Reverse 5′-AAAGAGATAACAGGAACGGA-3′; NQO1: Forward 5′-AAGGAGTTCCGGCTGTGATG-3′, Reverse 5′-TGCGAAATGGCAACAACGAA-3′; TLR4: Forward 5′-CACTTTATTCAGAGCCGTTG-3′, Reverse 5′-CCTTAGCTGATTTGGTCGAA-3′; NF-κB: Forward 5′-TATTGATGGCTGAGCAGCAG-3′, Reverse 5′-GGAGCTGTTGAATCCTGGAC-3′.

### 2.8. Statistical Analysis

The data was organized using WPS Office 2021, and SPSS 25.0 was employed to conduct significance and multiple comparisons of the experimental data.

## 3. Results

### 3.1. Isolation of a Structurally Uniform Neutral Polysaccharide from D. moschatum

**Figure 1:**
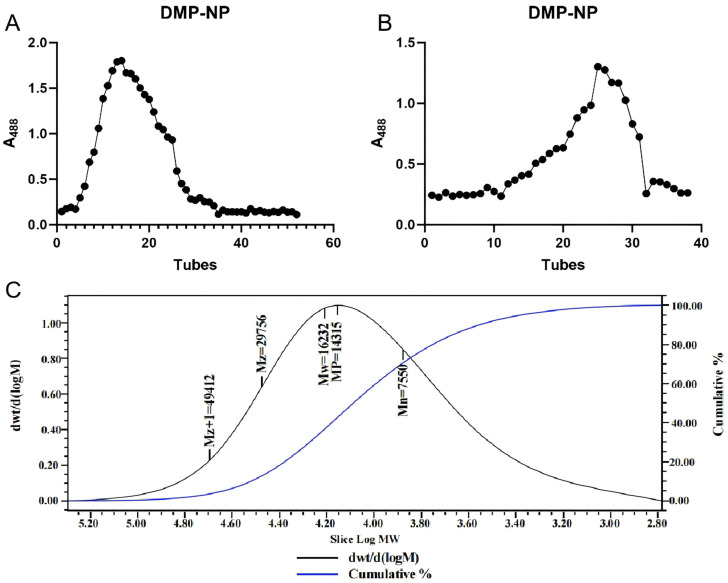
Isolation and purification of polysaccharides from *D. moschatum*. Wash elution curve of DMP-NP on DEAE Sepharose Fast Flow anion exchange column (**A**); Purification curve of DMP-NP1 on Sepharose 6 Fast Flow column (**B**); GPC chromatogram of DMP-NP polysaccharide (**C**).

The crude extract from *Dendrobium moschatum* was first fractionated by anion-exchange chromatography, a reliable method for separating neutral and acidic polysaccharides [12]. This yielded a neutral fraction (DMP-NP), whose elution profile displayed a single, well-defined peak, indicating initial purity (Figure 1A). The collected fraction was concentrated and freeze-dried, then subjected to further purification on a Sepharose 6 Fast Flow column. The final purified fraction, DMP-NP1, exhibited a homogeneous single peak (Figure 1B).

Gel permeation chromatography (GPC) is widely applied for the molecular weight analysis of high-MW soluble biopolymers such as proteins and polysaccharides [13]. However, the inherent structural heterogeneity and potential isomerism of polysaccharides often limit the resolution achievable by GPC alone [14]. Combining GPC with multi-angle laser light scattering (MALLS) overcomes this limitation, enabling accurate characterization of high-MW polysaccharides [15].

GPC-MALLS analysis determined the weight-average molecular mass of DMP-NP to be 16.2 kDa (Figure 1C). The symmetric, unimodal peak shape further confirmed the high molecular homogeneity of the purified polysaccharide.

### 3.2. Purification Enhances Polysaccharide Purity and Yields a Novel Glucose-Rich Heteropolysaccharide from D. moschatum

To gain deeper insights into the chemical composition of *D. moschatum* polysaccharides and their influence on physical, chemical, and biological properties, the total sugar, protein, and polyphenol contents of crude polysaccharides (DMP), neutral polysaccharides (DMP-NP), and purified neutral polysaccharides (DMP-NP1) were analyzed. The monosaccharide composition was determined using PMP derivatization followed by high-performance liquid chromatography (HPLC) [16].

The results showed that the polysaccharide contents of DMP, DMP-NP, and DMP-NP1 were 40.1%, 70.9%, and 92.7%, respectively. The total protein contents were 12.3%, 6.7%, and 1.4%, while the total polyphenol contents were relatively low at 2.4%, 1.7%, and 1.2%, respectively. PMP-HPLC analysis revealed that *D. moschatum* polysaccharides are typical heteropolysaccharides, primarily composed of glucose and mannose, which accounted for 96.39% of the total content. The molar ratio of monosaccharides was as follows: glucose–mannose–galactose–fucose–rhamnose = 78.54:19.11:1.59:0.53:0.23, with a glucose-to-mannose ratio of 4.1:1.

### 3.3. Infrared Spectroscopy Reveals Novel Structural Features of Dendrobium Neutral Polysaccharides

**Figure 2:**
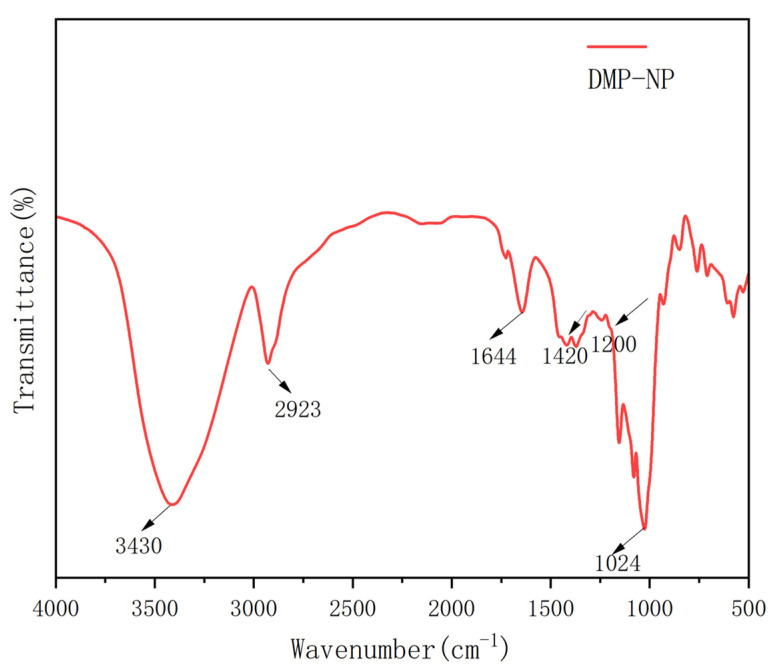
FT-IR spectrum of DMP-NP1.

To further analyze the chemical structure and functional groups of Dendrobium polysaccharides, we conducted infrared spectroscopy. This technique provides information on the absorption characteristics of different functional groups in molecules, helping reveal the chemical composition and structural features of polysaccharides. The FT-IR spectrum of DMP-NP1 is shown in Figure 2. The FT-IR spectrum exhibited characteristic polysaccharide absorptions [17], including a broad band at 3430–3000 cm^−1^ (O–H/N–H stretching) and a distinct peak at 2923 cm^−1^ (C–H stretching) [17]. These collective features confirmed the polysaccharidic nature of the purified neutral fraction from *Dendrobium*. Additional key features included an absorption at 1644 cm^−1^, indicative of C=O stretching in N-acetyl groups, and bands in the 1420–1200 cm^−1^ region, assigned to C–H bending vibrations [18]. Furthermore, the prominent peak at 1024 cm^−1^ is characteristic of C–O–C stretching in pyranose rings [19].

### 3.4. Structural Characterization of D. moschatum Polysaccharides Using NMR Spectroscopy

**Figure 3:**
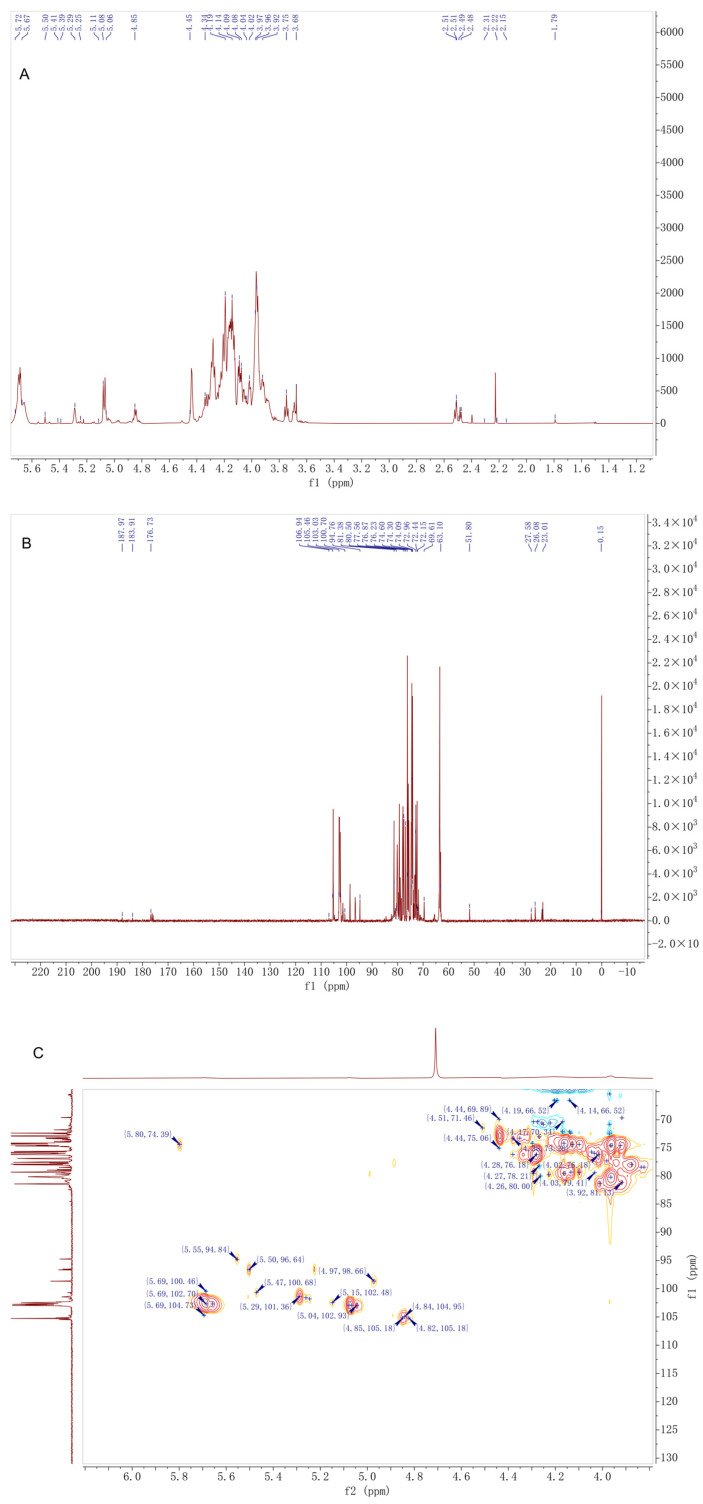
Structural characterization of *D. moschatum* polysaccharides (DMP-NP1): (**A**) ^1^H-NMR, (**B**) ^13^C-NMR and (**C**) HSQC spectra of DFM-NP1.

As shown in Figure 3 and Table 1. The ^1^H NMR spectrum displayed eight anomeric proton signals in the region of δ 4.45–5.72 ppm. Among these, signals at δ 5.69, 5.29, 5.08, 5.06, and 4.97 ppm were characteristic of α-configured anomeric protons, while those at δ 4.85, 4.84, and 4.45 ppm were assigned to β-configured anomeric protons. Among the α-configured signals, those at δ 5.29 and 5.08 ppm showed the highest intensities, suggesting they correspond to the major glucose residues. The three strong absorption peaks in the δ 2.15–2.23 ppm region were attributed to the methyl protons of acetyl groups, confirming the presence of O-acetylation in DMP-NP1. The continuous multiplet in the δ 3.64–4.19 ppm region corresponded to the sugar ring backbone protons, indicating a glucose-mannan type heteropolysaccharide.

The ^13^C NMR spectrum revealed six anomeric carbon signals (δ 96.64–105.46 ppm), which were assigned as follows: δ 105.46 and 105.18 ppm to β-anomeric carbons; δ 102.93 and 102.70 ppm to α-anomeric carbons of glucose; and δ 98.66 and 96.64 ppm to α-anomeric carbons of mannose. The acetyl carbonyl carbon signal at δ 176.73 ppm and the methyl carbon signal at δ 23.01 ppm further confirmed the presence of acetylation. Among the backbone carbon signals in the δ 62.0–90.03 ppm region, δ 81.35 and 81.38 ppm were assigned to carbons at the 1→4 glycosidic linkage sites, while the unique signal at δ 90.03 ppm was speculated to correspond to a special substitution at the C3 position of glucose residues, representing one of its distinctive structural features.

The HSQC spectrum, through direct hydrogen–carbon coupling signals, clarified the δH/δC correlations of major sugar residues, such as →4)-α-Glcp-(1→ at δH 5.29/δC 102.93 and →4,6)-α-Glcp-(1→ at δH 4.97/δC 98.66. The strong coupling signals of acetyl groups at δH 2.15–2.23/δC 23.01 indicated a uniform degree of acetylation substitution. HMBC correlations confirmed that the glycosidic linkage pattern of DMP-NP1 is dominated by 1→4 linkages, accompanied by a minor proportion of 1→6 branched linkages. The acetylation modifications were located at the C2 and C3 positions of mannose residues, showing a regular substitution pattern.

In summary, DMP-NP1 is a heteropolysaccharide primarily constituted of α-glucose and β-mannose residues interconnected by 1→4 glycosidic bonds to form the backbone. The structure is further characterized by the presence of occasional 1→6 branches, O-acetylation modifications, and unique substitutions on specific sugar residues. These structural characteristics provide a foundation for subsequent studies on its bioactivity.

Based on 1D/2D NMR and FT-IR analyses, DMP-NP1 can be proposed as a moderately branched glucan–mannan comprising a predominately α-1,4-linked D-Glcp backbone with occasional β-1,6-linked D-Manp side chains. Moderate O-acetylation was assigned to the C2/C3 positions of mannose residues, as evidenced by ^1^H/^13^C signals consistent with acetyl methyl and carbonyl groups and the FT-IR ester absorption at ~1644 cm^−1^. Terminal galactose, fucose and rhamnose residues were present at low abundance. Confirmation of linkage proportions and precise acetylation sites requires methylation-GC-MS and additional 2D NMR experiments.

### 3.5. Anti-Inflammatory Activity of D. moschatum Polysaccharides

**Figure 4:**
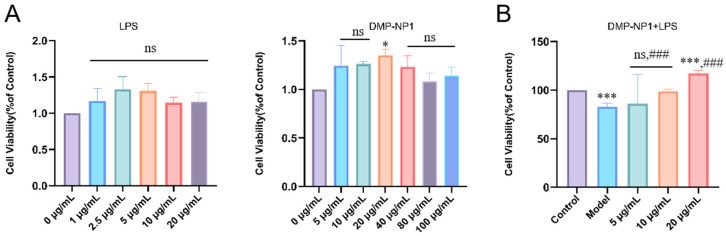
Cytotoxicity test. The effects of LPS and DMP-NP1 on cell viability (**A**); Effect of LPS on cell viability after treatment with different concentrations of DMP-NP1 (**B**). (n = 5) (Compared with the control group, * *p* < 0.05, *** *p* < 0.001, ns represents no significant difference; Compared with the model group, ### *p* < 0.001, ns represents no significant difference).

Research shows that LPS can induce pro-inflammatory factor secretion, triggering oxidative stress and increasing reactive oxygen species (ROS) levels. Excessive ROS damages cell structure and function. We used IPEC-J2 cells to create an LPS-induced inflammation model to study how *Dendrobium moschatum* neutral polysaccharide (DMP-NP1) prevents and treats cellular inflammation. Cells were treated with different DMP-NP1 concentrations, and viability was measured using CCK-8. As shown in Figure 4, at 0–100 μg/mL (0–6.16 μM), DMP-NP1 showed no toxicity to IPEC-J2 cells. Viability increased gradually between 0 and 20 μg/mL (0–1.23 μM) but declined at higher concentrations, so we set doses at 5 (low, 0.31 μM), 10 (medium, 0.62 μM), and 20 μg/mL (high, 1.23 μM).

To provide a potency estimate consistent with the observed activation-like response, we fitted a four-parameter logistic (4PL) model to the viability data and report an EC_50_ ≈ 5.94 µg/mL (4PL point estimate). We emphasize that this EC_50_ should be interpreted as an approximate potency indicator because the fitted parameters are subject to uncertainty arising from the limited number of concentration points and inter-replicate variability.

#### 3.5.1. Preventive Effects of D. moschatum Polysaccharides on LPS-Induced Inflammation in IPEC-J2 Cells

**Figure 5:**
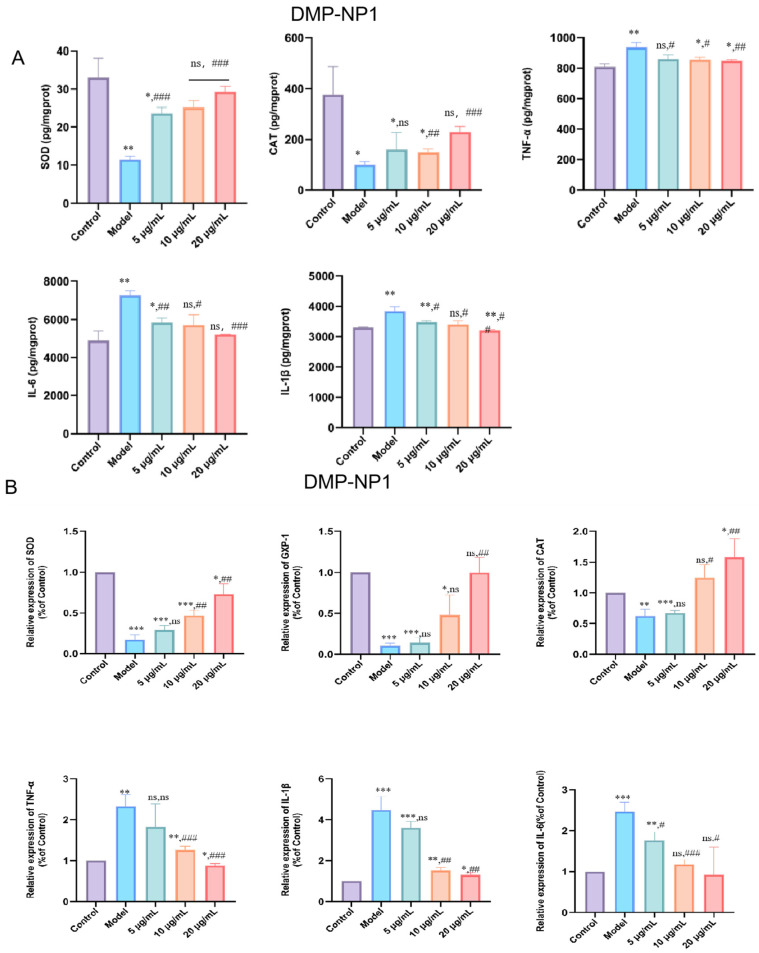
Effects of DMP-NP1 on the contents of CAT, SOD, TNF-α, IL-1β and IL-6 in IPEC-J2 cells (**A**); Effect of DMP-NP1 on the expression of *CAT*, *SOD*, *GXP-1*, *TNF-α*, *IL-1β*, *IL-6* (**B**). (n = 5) (IPEC-J2 cells were pretreated with DMP-NP1 (5, 10, 20 μg/mL) for 24 h, followed by exposure to LPS (20 μg/mL) for 24 h, control cells received no treatment, and the model group was exposed to LPS alone.) (Compared with the control group, * *p* < 0.05, ** *p* < 0.01, *** *p* < 0.001, ns represents no significant difference; Compared with the model group, # *p* < 0.05, ## *p* < 0.01, ### *p* < 0.001, ns represents no significant difference).

LPS stimulation markedly decreased SOD (*p* < 0.01) and CAT (*p* < 0.05) activities in IPEC-J2 cells, accompanied by significant increases in IL-1β, IL-6, and TNF-α levels (*p* < 0.01), indicating oxidative stress and a pronounced inflammatory response (Figure 5A). Pretreatment with DMP-NP1 significantly improved cell status in a dose-dependent manner. Specifically, TNF-α production was reduced at 0.31 and 0.62 μM (*p* < 0.05) and further suppressed at 1.23 μM (*p* < 0.01). IL-1β levels were significantly decreased at 0.31 and 0.62 μM (*p* < 0.05) and showed the greatest reduction at 1.23 μM (*p* < 0.01). IL-6 remained unchanged at 0.31 μM but was reduced at 0.62 μM (*p* < 0.05) and reached the lowest level at 1.23 μM (*p* < 0.001).

At the transcriptional level, CAT, SOD, and GPX1 mRNA expression were markedly suppressed by LPS (*p* < 0.01). DMP-NP1 treatment restored expression, with CAT and SOD significantly upregulated at 1.23 μM (*p* < 0.01, *p* < 0.001), while GPX1 was significantly increased only at 1.23 μM (*p* < 0.01) (Figure 5B).

Quantitative calculation shows that the EC_50_ for the enhancement of SOD and CAT activity was 0.68 µM and 0.79 µM, respectively. The IC_50_ for the inhibition of TNF-α, IL-6, and IL-1β secretion was 0.70 µM, 0.75 µM, and 0.72 µM, respectively. At the highest concentration tested (1.23 µM), DMP-NP1 reduced the levels of TNF-α, IL-6, and IL-1β by 63.2%, 65.8%, and 67.5% compared to the LPS model group.

Collectively, these results demonstrate that *D. moschatum* polysaccharides exert preventive effects against LPS-induced oxidative and inflammatory injury in IPEC-J2 cells by enhancing antioxidant enzyme activity and attenuating pro-inflammatory cytokine expression.

#### 3.5.2. Therapeutic Effect of D. moschatum Polysaccharides on LPS-Induced Inflammation in IPEC-J2 Cells

To evaluate the therapeutic potential of *Dendrobium moschatum* polysaccharides, an LPS-induced inflammatory model was established in IPEC-J2 cells, followed by treatment with the polysaccharide fraction. Oxidative stress levels and the expression of inflammation-related factors were then assessed.

**Figure 6:**
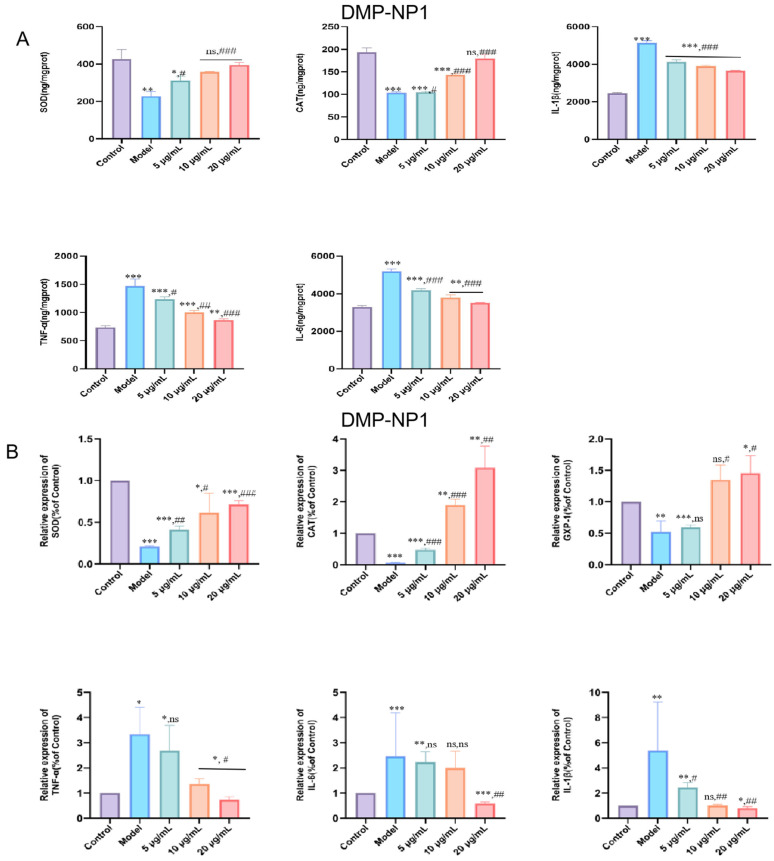
Effects of DMP-NP1 on CAT, SOD, TNF-α, IL-1β and IL-6 contents in IPEC-J2 inflammatory cells (**A**); Effect of DM-NP1 on *CAT*, *SOD*, *GXP-1*, *TNF-α*, *IL-1β*, and *IL-6* expression in IPEC-J2 inflammatory (**B**). (n = 5) (IPEC-J2 cells were first exposed to LPS (20 μg/mL) for 24 h to induce an inflammatory response, followed by co-incubation with DMP-NP1 at final concentrations of 0.31, 0.62, or 1.23 μM for an additional 24 h) (Compared with the control group, * *p* < 0.05, ** *p* < 0.01, *** *p* < 0.001, ns represents no significant difference; Compared with the model group, # *p* < 0.05, ## *p* < 0.01, ### *p* < 0.001, ns represents no significant difference).

Compared with the control group, LPS stimulation significantly elevated intracellular oxidative stress as well as the protein levels of TNF-α, IL-1β, and IL-6, whereas treatment with *D. moschatum* polysaccharides markedly reduced the levels of these three inflammatory factors in a concentration-dependent manner (Figure 6A).

These findings were further confirmed by q-PCR analysis. The results showed that CAT, SOD, and GPX-1 expression increased progressively with rising polysaccharide concentrations (Figure 6B), suggesting that *D. moschatum* polysaccharides possess potent antioxidant activity by upregulating antioxidant enzyme expression to counteract oxidative stress. In addition, the expression of inflammatory factors in IPEC-J2 cells was further examined following DMP-NP1 treatment. TNF-α (*p* < 0.05), IL-1β (*p* < 0.01), and IL-6 (*p* < 0.001) were significantly upregulated in the LPS model compared with the control, confirming that LPS triggered a pronounced inflammatory response. DMP-NP1 treatment downregulated TNF-α, IL-1β, and IL-6 gene expression in a dose-dependent manner, indicating that *D. moschatum* polysaccharides alleviate inflammation-related damage by suppressing pro-inflammatory mediators.

Quantitative analyses yielded EC_50_ values of 0.82 µM and 1.01 µM for the enhancement of SOD and CAT activity, respectively. The corresponding IC_50_ values for the inhibition of TNF-α, IL-6, and IL-1β secretion were 0.75 µM, 0.87 µM, and 0.83 µM. At the highest concentration tested (1.23 µM), DMP-NP1 reduced the levels of TNF-α, IL-6, and IL-1β by 41.7%, 32.8%, and 30.3% compared to the LPS model group.

In summary, DMP-NP1 dose-dependently restored antioxidant capacity and suppressed the expression of pro-inflammatory mediators. The precise molecular targets, however, such as direct inhibition of the NF-κB pathway or activation of Nrf2 signaling, represent key subjects for future investigation.

### 3.6. DMP-NP1 Enhances Antioxidant Defense via Modulation of the Keap1/Nrf2/HO-1/NQO1 Pathway in IPEC-J2 Cells

**Figure 7:**
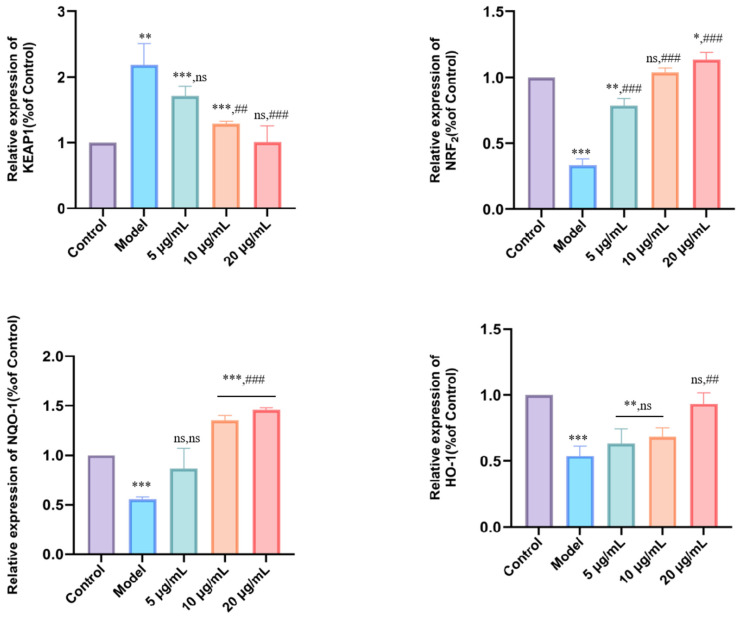
Effect of DMP-NP1 on genes related to oxidative stress pathway. (n = 5) (Compared with the control group, * *p* < 0.05, ** *p* < 0.01, *** *p* < 0.001, ns represents no significant difference; Compared with the model group, ## *p* < 0.01, ### *p* < 0.001, ns represents no significant difference).

The KEAP1/Nrf2-ARE pathway constitutes a vital intracellular defense system against oxidative stress. Activation of this pathway initiates with the dissociation of Nrf2 from its cytosolic repressor KEAP1, enabling Nrf2 translocation into the nucleus. There, it binds to antioxidant response elements (ARE) to drive the expression of downstream cytoprotective genes. To elucidate the regulatory mechanism underlying the antioxidant activity of DMP-NP1 in LPS-induced IPEC-J2 inflammatory cells, we examined the mRNA expression levels of *Keap1*, *Nrf2*, *NQO1*, and *HO-1*, as well as their regulatory relationship with downstream antioxidant enzymes such as SOD1, GXP1, and CAT (Figure 7).

Quantitative analysis revealed that LPS challenge (model group) significantly up-regulated *Keap1* expression (*p* < 0.01), while concurrently down-regulating the mRNA levels of *Nrf2*, *NQO1*, and *HO-1* (*p* < 0.01) compared to the control. These alterations indicate that increased *Keap1* expression and the concomitant suppression of *Nrf2*, *NQO1*, and *HO-1* contribute to enhanced oxidative stress in IPEC-J2 cells. Treatment with DMP-NP1 at 5 μg/mL (0.31 μM) did not significantly alter Keap1 expression, whereas 10 μg/mL and 20 μg/mL (0.62 and 1.23 μM) significantly downregulated Keap1 (*p* < 0.01). Notably, *Nrf2* expression was robustly upregulated at all tested concentrations of DMP-NP1 0.31, 0.62 and 1.23 μM, *p* < 0.001). For *NQO1*, no significant change was observed at 0.31 μM, but a highly significant increase occurred at 0.62 and 1.23 μM (*p* < 0.001). Similarly, *HO-1* expression remained unchanged at 0.31 μM, yet was significantly elevated at 0.62 and 1.23 μM (*p* < 0.01).

In summary, the inflammatory model was characterized by *Keap1* up-regulation coupled with suppression of the *Nrf2* pathway genes, thereby promoting oxidative stress and inflammation. In contrast, DMP-NP1 effectively suppressed Keap1 expression while markedly enhancing the expression of *Nrf2*, *NQO1*, and *HO-1*. Collectively, these results demonstrate that DMP-NP1 alleviates oxidative stress at the transcriptional level by suppressing *Keap1* and activating the Nrf2/HO-1/NQO1 signaling axis.

### 3.7. DMP-NP1 Effectively Suppresses TLR4/NF-κB Activation in IPEC-J2 Cells

**Figure 8:**
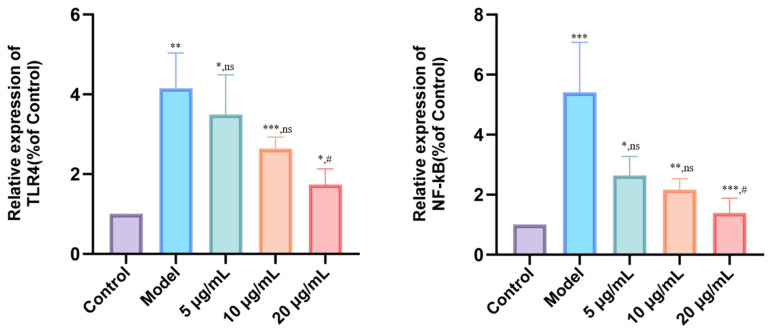
Effect of DMP-NP1 on anti-inflammatory pathway genes. (n = 5) (Compared with the control group, * *p* < 0.05, ** *p* < 0.01, *** *p* < 0.001, ns represents no significant difference; Compared with the model group, # *p* < 0.05, ns represents no significant difference).

To elucidate the anti-inflammatory mechanism of DMP-NP1, we analyzed the expression of key genes in the TLR4/NF-κB signaling pathway and their subsequent impact on the pro-inflammatory cytokines TNF-α, IL-1β, and IL-6 in LPS-induced IPEC-J2 cells. The results showed that, compared with the control group, the model group exhibited a significant upregulation of TLR4 mRNA (*p* < 0.01) and a marked increase in NF-κB mRNA expression (*p* < 0.001) (Figure 8). Similarly, no significant differences in NF-κB expression were observed in the 5 μg/mL (0.31 μM) and 10 μg/mL (0.62 μM) groups, while treatment with 20 μg/mL (1.23 μM) DMP-NP1 significantly downregulated NF-κB mRNA expression (*p* < 0.05).

These results demonstrate that LPS-induced inflammation is mediated through the up-regulation of *TLR4* and *NF-κB*, an effect that was counteracted by DMP-NP1 via transcriptional downregulation of these key signaling components. Notably, the inhibitory effect was most pronounced at the 20 μg/mL (1.23 μM), highlighting the dose-dependent anti-inflammatory activity of DMP-NP1 via modulation of the TLR4/NF-κB signaling pathway.

### 3.8. Correlation Analysis Between Inflammatory and Oxidative Stress Signaling Pathways

**Figure 9:**
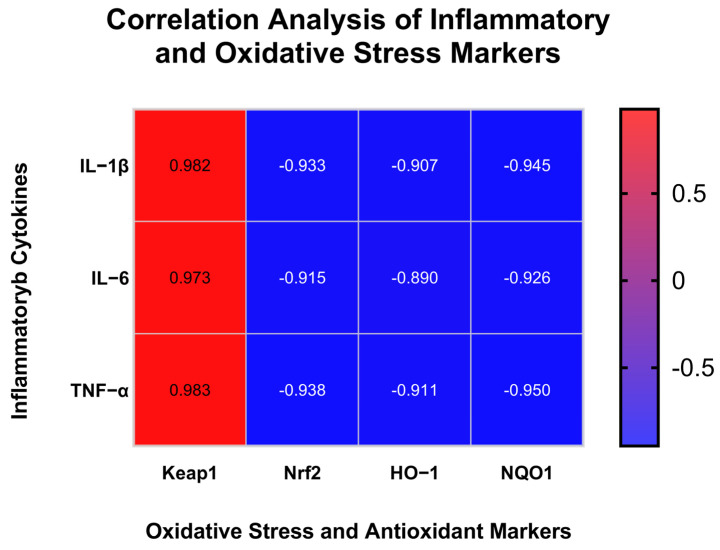
Correlation Analysis of Inflammatory and Oxidative Stress Markers.

To investigate the relationship between inflammation and oxidative stress signaling, Pearson correlation analysis was performed between cytokine protein levels (IL-1β, IL-6, TNF-α) and the mRNA expression of antioxidant-related genes (*Keap1*, *Nrf2*, *HO-1*, *NQO1*). The Pearson correlation matrix between inflammatory cytokines and antioxidant-related gene expression is shown in Figure 9. We found IL-1β, IL-6, and TNF-α levels were strongly and positively correlated with Keap1 expression (r = 0.982–0.983, *p* < 0.01), whereas they were negatively correlated with Nrf2, HO-1, and NQO1 expression (r = −0.89 to −0.95, *p* < 0.05). These results suggest that DMP-NP1 may alleviate inflammation through the suppression of Keap1 and concomitant activation of the Nrf2/ARE pathway.

## 4. Discussion

### 4.1. Monosaccharide Composition of Dendrobium Neutral Polysaccharides and Its Impact on Polysaccharide Bioactivity

Analysis of a polysaccharide’s monosaccharide composition is fundamental for distinguishing heteropolysaccharides from homopolysaccharides [19]. Previous studies have shown that the structural characteristics of plant-derived polysaccharides, including their monosaccharide composition, are closely associated with their anti-inflammatory and intestinal barrier–protective activities [20].Pre-column derivatization high-performance liquid chromatography (HPLC) is routinely employed for this analysis [21]. For example, polymers such as starch and cellulose, which consist of a single monosaccharide species, are classified as homopolysaccharides [22]. By contrast, Dendrobium polysaccharides typically contain multiple monosaccharides (e.g., glucose, mannose, galactose, rhamnose, and fucose) and are therefore heteropolysaccharides [23]. In our study, we demonstrate that the polysaccharide derived from *D. moschatum* is predominantly glucose-based, thereby designating it as a glucose-rich heteropolysaccharide. The presence of minor monosaccharides like mannose and fucose may contribute to the observed antioxidant and anti-inflammatory effects, though their specific roles warrant further study.

### 4.2. D. moschatum Neutral Polysaccharides Exhibit Significant Anti-Inflammatory Activity

Inflammatory responses are a crucial element of the innate immune system. While organisms activate self-regulatory immune feedback upon external stimulation, an excessive response can lead to tissue damage [24]. During inflammation, cells simultaneously produce anti-inflammatory and pro-inflammatory cytokines, with IL-1β, IL-6, and TNF-α serving as primary pro-inflammatory mediators [25]. The activation of the TLR4/MyD88/NF-κB signaling cascade—triggered by conditions such as colitis or LPS binding to TLR4—promotes the release of pro-inflammatory cytokines, thereby amplifying the inflammatory response [26].

Previous studies have demonstrated that Acorus polysaccharides can downregulate the gene expression of TLR4 and NF-κB, effectively suppressing pro-inflammatory cytokine secretion and ameliorating inflammatory symptoms [27]. Our findings are consistent with these reports: while LPS treatment upregulated TLR4 and NF-κB gene expression, *D. moschatum* polysaccharides inhibited this pathway by downregulating TLR4 and NF-κB, which in turn reduced the expression of downstream pro-inflammatory cytokines and mitigated inflammation [27].

Additionally, *Dendrobium huoshanense* polysaccharides have been shown to retain their high-molecular-weight form within the gut, enabling them to bind to TLR4 receptors on the surface of intestinal epithelial cells [28]. This binding lowers the levels of IL-6, MCP-1, and CINC-1, thereby modulating the mucosal immune response and exerting anti-inflammatory effects. Notably, the molecular weight of *D. huoshanense* polysaccharides used in that study was approximately 17.8 kDa, which is comparable to the molecular weight of the *D. moschatum* polysaccharides characterized here.

Notably, our correlation analysis unveiled a highly significant positive relationship between pro-inflammatory cytokines (IL-1β, IL-6, TNF-α) and *Keap1* expression, and a negative relationship with Nrf2 and its downstream genes. This finding provides a crucial mechanistic link between the two major signaling pathways investigated in this study. It strongly suggests that the antioxidant Nrf2 pathway and the inflammatory NF-κB pathway are co-regulated in our model, and that DMP-NP1 likely alleviates inflammation not only by directly targeting TLR4/NF-κB signaling but also indirectly through the potentiation of the cellular antioxidant defense system via Keap1/Nrf2 activation.

### 4.3. D. moschatum Neutral Polysaccharides Exert Significant Protective Effects Against Inflammation in Intestinal Epithelial Cells

Polysaccharides obtained from long-beam gold coin fungus through enzymatic hydrolysis (EnMPS) and acid hydrolysis (Ac-MPS) exhibit therapeutic potential against LPS-induced acute lung injury [29]. Their study revealed that these polysaccharides act via two principal mechanisms: on one hand, they enhance the serum activities of superoxide dismutase (SOD), glutathione peroxidase (GSH-Px), catalase (CAT), and total antioxidant capacity (T-AOC); on the other, they effectively reduce the levels of pro-inflammatory cytokines such as TNF-α, IL-1β, and IL-6, thereby providing both antioxidant and anti-inflammatory protection.

Similarly, *Tassel Dendrobium* has been shown to alleviate dextran sulfate sodium-induced colitis in mice by modulating the Nrf2/NF-κB signaling pathway, highlighting the crosstalk between these pathways in regulating oxidative stress and inflammation [30]. In addition, previous work showed that oral administration of *Tassel Dendrobium* polysaccharides ameliorates ulcerative colitis by regulating inflammatory cytokine secretion, boosting antioxidant enzyme activity, and modulating both the intestinal microbiota and gut permeability.

In the present study, we investigate the dual protective effects of *D. moschatum* polysaccharides against oxidative stress and inflammation in LPS-stimulated intestinal epithelial cells. Our results demonstrate that these polysaccharides act through a concerted modulation of both the Nrf2/HO-1 and TLR4/NF-κB signaling pathways. Quantitative results clearly demonstrate that DMP-NP1 possesses potent, dose-dependent antioxidant and anti-inflammatory effects in both preventive and therapeutic contexts. A consistent and noteworthy finding is that the preventive effects were consistently more potent (lower EC_50_/IC_50_ values) and efficacious (greater maximal inhibition) than the therapeutic effects. This suggests that pre-activation of cellular defense pathways by DMP-NP1 provides superior protection against LPS-induced intestinal epithelial injury compared to intervention after inflammatory cascade initiation.

### 4.4. Structural Features of DMP-NP1 Suggest Potential Mechanisms for Antioxidant and Anti-Inflammatory Functions

The structural characterization of *Dendrobium moschatum* neutral polysaccharide (DMP-NP1) by one- and two-dimensional NMR spectroscopy revealed a glucose–mannan heteropolysaccharide with distinctive substitution motifs. The ^1^H and ^13^C NMR spectra demonstrated that the polymer backbone is primarily composed of α-Glc and β-Man residues, connected mainly through 1→4 glycosidic bonds, with a minor proportion of 1→6 linkages. Several well-resolved acetyl signals were observed, and HMBC analysis indicated that O-acetylation occurs predominantly at the C2 and C3 positions of mannose residues, suggesting a regular substitution pattern. In addition, a unique resonance at δC ~90 ppm was tentatively assigned to a substituted C3 of glucose, which may represent a novel structural feature not commonly described in plant-derived glucomannans.

O-Acetylation critically influences the physicochemical and functional properties of polysaccharides. Papatheodorou et al. (2023) reported that acetylation at C2 and C3 of β-mannans strongly influences solubility and conformational flexibility, thereby affecting their role in plant cell walls and water interactions [31]. Similarly, the regular acetylation observed in DMP-NP1 may enhance its resistance to enzymatic or chemical degradation and alter its hydrophilic–hydrophobic balance, which could be relevant for its biological activity. Bhullar et al. (2022) further demonstrated in konjac glucomannan that acetylation distribution modulates gelling capacity and processing properties, highlighting the functional importance of substitution patterns [32]. The comparable substitution in DMP-NP1 suggests potential implications for its stability and interaction with biomolecules.

The high proportion of α-Glcp in the backbone of DMP-NP1 also differentiates it from typical plant glucomannans, which are generally dominated by β-mannose units. Casillo et al. (2021) emphasized that branching and acetylation substantially influence the biological properties of mannan-based polysaccharides, such as immunomodulation and resistance to degradation [33]. Therefore, the confluence of α-Glcp dominance, minor 1→6 branching, and regular O-acetylation in DMP-NP1 is likely to confer distinctive conformational and biological properties relative to other reported glucomannans. 

Taken together, the structural features of DMP-NP1—an α-Glcp/β-Man heteropolysaccharide with a predominantly 1→4 backbone, limited 1→6 branching, and regular O-acetylation at mannose C2/C3—suggest a molecule with potential functional advantages. The presence of a distinctive C3 substitution signal in glucose residues may represent a novel modification, expanding current knowledge of structural diversity in polysaccharides from the genus Dendrobium. These structural insights establish a foundation for elucidating the relationship between specific chemical modifications and bioactivity, particularly in the context of antioxidant, anti-inflammatory, or immunomodulatory functions that have been associated with acetylated plant polysaccharides.

### 4.5. Structure–Activity Relationship and Mechanistic Insights

The present study establishes a connection between the anti-inflammatory and antioxidant effects of *Dendrobium moschatum* neutral polysaccharide (DMP-NP1) and its characterized structural features. The purified material is characterized by a moderate average molecular weight (~16.2 kDa), a glucose-rich α-1,4 backbone with intermittent β-1,6 mannose branches, and moderate O-acetylation at mannose C2/C3. Such a moderately branched, partially acetylated glucan–mannan architecture is consistent with structural motifs that have been repeatedly associated with immunomodulatory and antioxidant activities of natural polysaccharides.

From a structure–activity relationship (SAR) perspective, several interrelated structural determinants plausibly explain the biological readouts observed here.

First, a moderate molecular weight (≈10–30 kDa) often balances mucosal retention and receptor accessibility—it is large enough to present multivalent binding epitopes to carbohydrate-binding receptors yet small enough to avoid excessive viscosity or steric hindrance that reduces bioavailability. Previous studies indicate that polysaccharides within this range tend to exhibit enhanced immunomodulatory and antioxidant properties [34,35,36].

Second, the presence of mannose residues serve as recognition motifs for C-type lectin receptors (such as the mannose receptor, MR, and DC-SIGN), which can interact with Toll-like receptor 4 (TLR4) complexes to shape downstream inflammatory signaling. This receptor-mediated modulation aligns with our observed downregulation of the TLR4/NF-κB pathway in DMP-NP1-treated cells [37,38,39]. Third, O-acetyl groups on mannose residues may influence hydrophobicity and spatial conformation, enhancing stability, receptor affinity, and cellular uptake. Acetylated polysaccharides have been shown to activate the Keap1/Nrf2/ARE signaling pathway, leading to upregulation of antioxidant enzymes such as HO-1 and NQO1. This is consistent with our data showing that DMP-NP1 increases *Nrf2* and its downstream gene expression while decreasing *Keap1* transcription [40].

Taken together, DMP-NP1 exhibits a dual regulatory effect: it suppresses the pro-inflammatory TLR4/NF-κB axis and activates the Keap1/Nrf2 antioxidant pathway. This bidirectional regulation is structurally enabled by its moderate molecular weight, α-1,4 glucan backbone, β-linked mannose branches, and partial O-acetylation.

The SAR insights presented here provide a mechanistic foundation for the rational design of polysaccharide-based therapeutics for inflammatory bowel disease (IBD). Future SAR-guided optimization could focus on controlling molecular weight and acetylation degree to fine-tune receptor recognition, antioxidant potency, and intestinal bioavailability.

### 4.6. Limitations of the Study

Although the present study provides strong evidence that *Dendrobium moschatum* neutral polysaccharide (DMP-NP1) exerts anti-inflammatory and antioxidant effects through modulation of the Keap1/Nrf2/ARE signaling pathway, several limitations should be acknowledged.

First, the protein expression of key pathway components (Keap1, Nrf2, HO-1, and NQO1) was not verified by Western blot due to experimental constraints; only mRNA levels were assessed, which may not fully reflect post-transcriptional regulation.

Second, oxidative stress was evaluated mainly by enzymatic activities (SOD, CAT) and related gene expression, while classical lipid peroxidation and thiol-based indicators such as MDA and GSH were not measured.

Third, this study utilized an in vitro model, and further validation using in vivo IBD models is warranted to confirm the therapeutic relevance and bioavailability of DMP-NP1.

Despite these limitations, the consistent biochemical and molecular data support the conclusion that DMP-NP1 alleviates inflammation by activating the Nrf2/ARE pathway and restoring redox homeostasis. Future studies will address these limitations through integrated protein-level validation, metabolite assays, and animal experiments.

## 5. Conclusions

This study demonstrates that the purified polysaccharide DMP-NP1 (16.23 kDa), isolated from *Dendrobium moschatum*, exhibits significant antioxidant and anti-inflammatory activities in LPS-challenged IPEC-J2 intestinal epithelial cells. Notably, DMP-NP1 exhibited no cytotoxicity and effectively attenuates the inflammatory response by enhancing the activity of key antioxidant enzymes (CAT and SOD), suppressing the secretion of pro-inflammatory cytokines (TNF-α, IL-1β, IL-6), and downregulating the expression of genes related to inflammation and the TLR4/NF-κB signaling pathway. Collectively, these findings highlight the potential of DMP-NP1, and polysaccharides from *D. moschatum* more broadly, as a therapeutic candidate for mitigating intestinal disorders associated with oxidative stress and inflammation.

## Figures and Tables

**Table 1:** Chemical shift assignment of DMP-NP1 (δ, ppm).

Residues	H1/C1	H2/C2	H3/C3	H4/C4	H5/C5	H6/C6
→4)-α-Glcp-(1→	5.29/102.93	3.64/74.60	3.97/90.03	3.64/81.35	3.85/76.18	3.93,3.77/62.80
Glcp-(1→)	5.08/102.70	3.59/74.30	3.70/72.96	3.44/72.44	3.81/76.18	3.93,3.77/62.60
→4,6)-α-Glcp-(1→)	4.97/98.66	3.64/74.60	3.69/72.15	3.60/81.38	3.77/77.56	3.90,3.94/70.57
→4)-β-Manp-(1→)	4.71/103.03	4.11/73.04	3.94/79.33	3.84/80.50	3.59/76.87	3.92,3.77/62.40
→4)-2-O-Ac-β-Manp-(1→) *	4.85/105.18	5.48/74.39	4.02/79.56	3.84/80.50	3.61/77.56	3.92,3.77/62.20
→4)-3-O-Ac-β-Manp-(1→) *	4.84/101.36	4.18/70.57	5.09/76.23	3.81/80.50	3.59/76.87	3.92,3.77/62.00
→4)-α-Manp	5.06/96.64	3.58/74.09	n.d./n.d.	n.d./n.d.	n.d./n.d.	n.d./n.d.
→4)-β-Glcp-(1→)	4.45/105.46	3.37/72.44	3.63/71.60	3.81/79.33	n.d./n.d.	3.93,3.77/62.00

“*”, denotes acetylated residues, including →4)-2-O-Ac-β-Manp-(1→ and →4)-3-O-Ac-β-Manp-(1→. The characteristic O-Ac (O–CO–CH_3_) methyl and carbonyl signals were observed at δ 2.20/23.1 (176.0), 2.19/23.1 (176.2), 2.16/23.1 (175.8), and 2.11/23.1 (175.5) in the HSQC and HMBC spectra, confirming acetyl substitution at O-2 and O-3 of β-Manp residues. n.d.: Not assigned.

## Data Availability

The original contributions presented in this study are included in the article. Further inquiries can be directed to the corresponding authors.
